# The Concise Guide to PHARMACOLOGY 2015/16: Nuclear hormone receptors

**DOI:** 10.1111/bph.13352

**Published:** 2015-12-09

**Authors:** Stephen PH Alexander, John A Cidlowski, Eamonn Kelly, Neil Marrion, John A Peters, Helen E Benson, Elena Faccenda, Adam J Pawson, Joanna L Sharman, Christopher Southan, Jamie A Davies, CGTP Collaborators

**Affiliations:** ^1^ School of Biomedical Sciences University of Nottingham Medical School Nottingham NG7 2UH UK; ^2^ National Institute of Environmental Health Sciences National Institutes of Health Department of Health and Human Services Research Triangle Park NC 27709 USA; ^3^ School of Physiology and Pharmacology University of Bristol Bristol BS8 1TD UK; ^4^ Neuroscience Division, Medical Education Institute, Ninewells Hospital and Medical School University of Dundee Dundee DD1 9SY UK; ^5^ Centre for Integrative Physiology University of Edinburgh Edinburgh EH8 9XD UK

## Abstract

The Concise Guide to PHARMACOLOGY 2015/16 provides concise overviews of the key properties of over 1750 human drug targets with their pharmacology, plus links to an open access knowledgebase of drug targets and their ligands (www.guidetopharmacology.org), which provides more detailed views of target and ligand properties. The full contents can be found at http://onlinelibrary.wiley.com/doi/10.1111/bph.13352/full. Nuclear hormone receptors are one of the eight major pharmacological targets into which the Guide is divided, with the others being: G protein‐coupled receptors, ligand‐gated ion channels, voltage‐gated ion channels, other ion channels, catalytic receptors, enzymes and transporters. These are presented with nomenclature guidance and summary information on the best available pharmacological tools, alongside key references and suggestions for further reading. The Concise Guide is published in landscape format in order to facilitate comparison of related targets. It is a condensed version of material contemporary to late 2015, which is presented in greater detail and constantly updated on the website www.guidetopharmacology.org, superseding data presented in the previous Guides to Receptors & Channels and the Concise Guide to PHARMACOLOGY 2013/14. It is produced in conjunction with NC‐IUPHAR and provides the official IUPHAR classification and nomenclature for human drug targets, where appropriate. It consolidates information previously curated and displayed separately in IUPHAR‐DB and GRAC and provides a permanent, citable, point‐in‐time record that will survive database updates.

## Conflict of interest

The authors state that there are no conflicts of interest to declare.

## Overview

Nuclear hormone receptors are specialised transcription factors with commonalities of sequence and structure, which bind as homo‐ or heterodimers to specific consensus sequences of DNA (response elements) in the promoter region of particular target genes. They regulate (either promoting or repressing) transcription of these target genes in response to a variety of endogenous ligands. Endogenous agonists are hydrophobic entities which, when bound to the receptor promote conformational changes in the receptor to allow recruitment (or dissociation) of protein partners, generating a large multiprotein complex.

Two major subclasses of nuclear receptors with identified endogenous agonists can be identified: steroid and non‐steroid hormone receptors. Steroid hormone receptors function typically as dimeric entities and are thought to be resident outside the nucleus in the unliganded state in a complex with chaperone proteins, which are liberated upon agonist binding. Migration to the nucleus and interaction with other regulators of gene transcription, including RNA polymerase, acetyltransferases and deacetylases, allows gene transcription to be regulated. Non‐steroid hormone receptors typically exhibit a greater distribution in the nucleus in the unliganded state and interact with other nuclear receptors to form heterodimers, as well as with other regulators of gene transcription, leading to changes in gene transcription upon agonist binding.

Selectivity of gene regulation is brought about through interaction of nuclear receptors with particular consensus sequences of DNA, which are arranged typically as repeats or inverted palindromes to allow accumulation of multiple transcription factors in the promoter regions of genes.

## Family structure

5958 1A. Thyroid hormone receptors

5959 1B. Retinoic acid receptors

5960 1C. Peroxisome proliferator‐activated receptors

5961 1D. Rev‐Erb receptors

5962 1F. Retinoic acid‐related orphans

5963 1H. Liver X receptor‐like receptors

5964 1I. Vitamin D receptor‐like receptors

5965 2A. Hepatocyte nuclear factor‐4 receptors

5966 2B. Retinoid X receptors

5967 2C. Testicular receptors

5968 2E. Tailless‐like receptors

5969 2F. COUP‐TF‐like receptors

5970 3B. Estrogen‐related receptors

5971 4A. Nerve growth factor IB‐like receptors

5972 5A. Fushi tarazu F1‐like receptors

5973 6A. Germ cell nuclear factor receptors

5974 0B. DAX‐like receptors

5975 Steroid hormone receptors

5975 3A. Estrogen receptors

5976 3C. 3‐Ketosteroid receptors

## 1A. Thyroid hormone receptors

### Overview

Thyroid hormone receptors (**TRs, nomenclature as agreed by the** **NC‐IUPHAR** **Subcommittee on Nuclear Hormone Receptors** [**41**]) are nuclear hormone receptors of the NR1A family, with diverse roles regulating macronutrient metabolism, cognition and cardiovascular homeostasis. TRs are activated by thyroxine (T_4_) and thyroid hormone (triiodothyronine). Once activated by a ligand, the receptor acts as a transcription factor either as a monomer, homodimer or heterodimer with members of the retinoid X receptor family. NH‐3 has been described as an antagonist at TRs with modest selectivity for TRβ[111].

**Table:** 

Nomenclature	Thyroid hormone receptor‐α	Thyroid hormone receptor‐β
Systematic nomenclature	NR1A1	NR1A2
HGNC, UniProt	THRA, P10827	THRB, P10828
Rank order of potency	triiodothyronine>T_4_	triiodothyronine>T_4_
Agonists	dextrothyroxine [20]	dextrothyroxine [20]
Selective agonists	–	sobetirome (p*K*_d_ 10.2) [27, 133]

### Comments

An interaction with integrin αVβ3 has been suggested to underlie plasma membrane localization of TRs and non‐genomic signalling [9].One splice variant, TRα_2_, lacks a functional DNA‐binding domain and appears to act as a transcription suppressor.

Although radioligand binding assays have been described for these receptors, the radioligands are not commercially available.

### Further Reading

Bianco AC. (2011) Minireview: cracking the metabolic code for thyroid hormone signaling. *Endocrinology* **152**: 3306‐11 [PMID:21712363]

Brent GA. (2012) Mechanisms of thyroid hormone action. *J. Clin. Invest.* **122**: 3035‐43 [PMID:22945636]

Flamant F *et al*. (2006) International Union of Pharmacology. LIX. The pharmacology and classification of the nuclear receptor superfamily: thyroid hormone receptors. *Pharmacol. Rev.* **58**: 705‐11 [PMID:17132849]

Pramfalk C *et al*. (2011) Role of thyroid receptor β in lipid metabolism. *Biochim. Biophys. Acta* **1812**: 929‐37 [PMID:21194564]

Sirakov M *et al*. (2011) The thyroid hormones and their nuclear receptors in the gut: from developmental biology to cancer. *Biochim. Biophys. Acta* **1812**: 938‐46 [PMID:21194566]

Tancevski I *et al*. (2011) Thyromimetics: a journey from bench to bed‐side. *Pharmacol. Ther.* **131**: 33‐9 [PMID:21504761]

## 1B. Retinoic acid receptors

### Overview

Retinoic acid receptors (**nomenclature as agreed by the** **NC‐IUPHAR** **Subcommittee on Nuclear Hormone Receptors** [**46**]) are nuclear hormone receptors of the NR1B family activated by the vitamin A‐derived agonists tretinoin (ATRA) and alitretinoin, and the RAR‐selective synthetic agonists TTNPB and adapalene. BMS493 is a family‐selective antagonist [47].

**Table:** 

Nomenclature	Retinoic acid receptor‐α	Retinoic acid receptor‐β	Retinoic acid receptor‐γ
Systematic nomenclature	NR1B1	NR1B2	NR1B3
HGNC, UniProt	RARA, P10276	RARB, P10826	RARG, P13631
Agonists	tretinoin (pEC_50_ 7.8) [26]	tretinoin (pEC_50_ 7.9) [26]	tretinoin (pEC_50_ 9.7) [26]
(Sub)family‐selective agonists	tazarotene (pEC_50_ 7.2) [26]	tazarotene (pEC_50_ 9.1) [26], adapalene (p*K*_i_ 7.5) [25]	tazarotene (pEC_50_ 7.4) [26], adapalene (p*K*_i_ 6.9) [25]
Selective agonists	BMS753 (p*K*_i_ 8.7) [53], Ro 40‐6055 (p*K*_d_ 8.2) [33], tamibarotene (pIC_50_ 6.9) [65, 108, 146]	AC261066 (pEC_50_ 7.9–8.1) [90], AC55649 (pEC_50_ 6.5–7.3) [90]	AHPN (p*K*_i_ 7.1) [25]
Selective antagonists	Ro 41‐5253 (pIC_50_ 6.3–7.2) [2, 70]	–	MM 11253 [77]

### Comments

Ro 41‐5253 has been suggested to be a PPARγ agonist [131]. LE135 is an antagonist with selectivity for RARα and RARβ compared with RARγ[85].

### Further Reading

Bour G *et al*. (2007) Protein kinases and the proteasome join in the combinatorial control of transcription by nuclear retinoic acid receptors. *Trends Cell Biol.* **17**: 302‐9 [PMID:17467991]

Duong V *et al*. (2011) The molecular physiology of nuclear retinoic acid receptors. From health to disease. *Biochim. Biophys. Acta* **1812**: 1023‐31 [PMID:20970498]

Germain P *et al*. (2006) International Union of Pharmacology. LX. Retinoic acid receptors. *Pharmacol. Rev.* **58**: 712‐25 [PMID:17132850]

Maden M. (2007) Retinoic acid in the development, regeneration and maintenance of the nervous system. *Nat. Rev. Neurosci.* **8**: 755‐65 [PMID:17882253]

Mark M *et al*. (2006) Function of retinoid nuclear receptors: lessons from genetic and pharmacological dissections of the retinoic acid signaling pathway during mouse embryogenesis. *Annu. Rev. Pharmacol. Toxicol.* **46**: 451‐80 [PMID:16402912]

## 1C. Peroxisome proliferator‐activated receptors

### Overview

Peroxisome proliferator‐activated receptors (**PPARs, nomenclature as agreed by the** **NC‐IUPHAR** **Subcommittee on Nuclear Hormone Receptors**[**101**]) are nuclear hormone receptors of the NR1C family, with diverse roles regulating lipid homeostasis, cellular differentiation, proliferation and the immune response. PPARs have many potential endogenous agonists [14, 101], including 15‐deoxy‐*Δ*^12,14^‐PGJ_2_, prostacyclin (PGI_2_), many fatty acids and their oxidation products, lysophosphatidic acid (LPA) [98], 13‐HODE, 15S‐HETE, Paz‐PC, azelaoyl‐PAF and leukotriene B4 (LTB_4_). Bezafibrate acts as a non‐selective agonist for the PPAR family [159]. These receptors also bind hypolipidaemic drugs (PPARα) and anti‐diabetic thiazolidinediones (PPARγ), as well as many non‐steroidal anti‐inflammatory drugs, such as sulindac and indomethacin. Once activated by a ligand, the receptor forms a heterodimer with members of the retinoid X receptor family and can act as a transcription factor. Although radioligand binding assays have been described for all three receptors, the radioligands are not commercially available. Commonly, receptor occupancy studies are conducted using fluorescent ligands and truncated forms of the receptor limited to the ligand binding domain.

**Table:** 

Nomenclature	Peroxisome proliferator‐activated receptor‐α	Peroxisome proliferator‐activated receptor‐β/δ	Peroxisome proliferator‐activated receptor‐γ
Systematic nomenclature	NR1C1	NR1C2	NR1C3
HGNC, UniProt	PPARA, Q07869	PPARD, Q03181	PPARG, P37231
Agonists	–	–	mesalazine (pIC_50_ 1.8) [126]
Selective agonists	GW7647 (pEC_50_ 8.2) [18, 19], CP‐775146 (pEC_50_ 7.3) [68], pirinixic acid (pEC_50_ 5.3) [159], gemfibrozil (pEC_50_ 4.2) [31], ciprofibrate	GW0742X (pIC_50_ 9) [50, 144], GW501516 (pEC_50_ 9) [113]	GW1929 (p*K*_i_ 8.8) [18], bardoxolone (Partial agonist) (p*K*_i_ 8) [153], rosiglitazone (p*K*_d_ 7.4) [59, 81, 165], rosiglitazone (p*K*_i_ 6.9) [88], troglitazone (pIC_50_ 6.3) [59, 165], pioglitazone (pIC_50_ 6.2) [59, 129, 165], troglitazone (p*K*_i_ 5.8) [8], ciglitazone (pEC_50_ 4.6) [59]
Selective antagonists	GW6471 (pIC_50_ 6.6) [162]	GSK0660 (pIC_50_ 6.5) [134]	T0070907 (p*K*_i_ 9) [78], GW9662 (Irreversible inhibition) (pIC_50_ 8.1) [79], CDDO‐Me (p*K*_i_ 6.9) [153]

### Comments

As with the estrogen receptor antagonists, many agents show tissue‐selective efficacy (*e.g*. [13, 110, 125]). Agonists with mixed activity at PPARα and PPARγ have also been described (*e.g* [35, 52, 163]).

### Further Reading

Huang JV *et al*. (2012) PPAR‐γ as a therapeutic target in cardiovascular disease: evidence and uncertainty. *J. Lipid Res.* **53**: 1738‐54 [PMID:22685322]

Michalik L *et al*. (2006) International Union of Pharmacology. LXI. Peroxisome proliferator‐activated receptors. *Pharmacol. Rev.* **58**: 726‐41 [PMID:17132851]

Michalik L *et al*. (2008) PPARs Mediate Lipid Signaling in Inflammation and Cancer. *PPAR Res* **2008**: 134059 [PMID:19125181]

Peters JM *et al*. (2012) The role of peroxisome proliferator‐activated receptors in carcinogenesis and chemoprevention. *Nat. Rev. Cancer* **12**: 181‐95 [PMID:22318237]

Pirat C *et al*. (2012) Targeting peroxisome proliferator‐activated receptors (PPARs): development of modulators. *J. Med. Chem.* **55**: 4027‐61 [PMID:22260081]

Varga T *et al*. (2011) PPARs are a unique set of fatty acid regulated transcription factors controlling both lipid metabolism and inflammation. *Biochim. Biophys. Acta* **1812**: 1007‐22 [PMID:21382489]

Youssef J *et al*. (2011) Peroxisome proliferator‐activated receptors and cancer: challenges and opportunities. *Br. J. Pharmacol.* **164**: 68‐82 [PMID:21449912]

## 1D. Rev‐Erb receptors

### Overview

Rev‐erb receptors (**nomenclature as agreed by the** **NC‐IUPHAR** **Subcommittee on Nuclear Hormone Receptors** [**7**]) have yet to be officially paired with an endogenous ligand, but are thought to be activated by heme.

**Table:** 

Nomenclature	Rev‐Erb‐α	Rev‐Erb‐β
Systematic nomenclature	NR1D1	NR1D2
HGNC, UniProt	NR1D1, P20393	NR1D2, Q14995
Endogenous agonists	heme (Selective) [122, 164]	heme (Selective) [122, 164]
Selective agonists	GSK4112 (pEC_50_ 6.4) [51], GSK4112 (pIC_50_ 5.6) [73]	–
Selective antagonists	SR8278 (pIC_50_ 6.5) [73]	–

### Further Reading

Benoit G *et al*. (2006) International Union of Pharmacology. LXVI. Orphan nuclear receptors. *Pharmacol. Rev.* **58**: 798‐836 [PMID:17132856]

Duez H *et al*. (2009) Rev‐erb‐alpha: an integrator of circadian rhythms and metabolism. *J. Appl. Physiol.* **107**: 1972‐80 [PMID:19696364]

Germain P *et al*. (2006) Overview of nomenclature of nuclear receptors. *Pharmacol. Rev.* **58**: 685‐704 [PMID:17132848]

Huang P *et al*. (2010) Structural overview of the nuclear receptor superfamily: insights into physiology and therapeutics. *Annu. Rev. Physiol.* **72**: 247‐72 [PMID:20148675]

Phelan CA *et al*. (2010) Structure of Rev‐erbalpha bound to N‐CoR reveals a unique mechanism of nuclear receptor‐co‐repressor interaction. *Nat. Struct. Mol. Biol.* **17**: 808‐14 [PMID:20581824]

Sladek FM. (2011) What are nuclear receptor ligands? *Mol. Cell. Endocrinol.* **334**: 3‐13 [PMID:20615454]

Yin L *et al*. (2010) Nuclear receptor Rev‐erbalpha: a heme receptor that coordinates circadian rhythm and metabolism. *Nucl Recept Signal* **8**: e001 [PMID:20414452]

## 1F. Retinoic acid‐related orphans

### Overview

Retinoic acid receptor‐related orphan receptors (ROR, **nomenclature as agreed by the** **NC‐IUPHAR** **Subcommittee on Nuclear Hormone Receptors** [**7**]) have yet to be assigned a definitive endogenous ligand, although RORα may be synthesized with a ‘captured’ agonist such as cholesterol[66, 67].

**Table:** 

Nomenclature	RAR‐related orphan receptor‐α	RAR‐related orphan receptor‐β	RAR‐related orphan receptor‐γ
Systematic nomenclature	NR1F1	NR1F2	NR1F3
HGNC, UniProt	RORA, P35398	RORB, Q92753	RORC, P51449
Endogenous agonists	cholesterol (Selective) [67, 115]	–	–
Selective agonists	7‐hydroxycholesterol [15], cholesterol sulphate [15, 67]	–	–

### Comments

tretinoin shows selectivity for RORβ within the ROR family [139]. RORα has been suggested to be a nuclear receptor responding to melatonin[158].

### Further Reading

Benoit G *et al*. (2006) International Union of Pharmacology. LXVI. Orphan nuclear receptors. *Pharmacol. Rev.* **58**: 798‐836 [PMID:17132856]

## 1H. Liver X receptor‐like receptors

### Overview

Liver X and farnesoid X receptors (LXR and FXR, **nomenclature as agreed by the** **NC‐IUPHAR** **Subcommittee on Nuclear Hormone Receptors**[**105**]) are members of a steroid analogue‐activated nuclear receptor subfamily, which form heterodimers with members of the retinoid X receptor family. Endogenous ligands for LXRs include hydroxycholesterols (OHC), while FXRs appear to be activated by bile acids.

**Table:** 

Nomenclature	Farnesoid X receptor	Farnesoid X receptor‐β	Liver X receptor‐α	Liver X receptor‐β
Systematic nomenclature	NR1H4	NR1H5	NR1H3	NR1H2
HGNC, UniProt	NR1H4, Q96RI1	NR1H5P, –	NR1H3, Q13133	NR1H2, P55055
Potency order	chenodeoxycholic acid>lithocholic acid, deoxycholic acid [93, 116]	–	20S‐hydroxycholesterol, 22R‐hydroxycholesterol, 24(S)‐hydroxycholesterol>25‐hydroxycholesterol, 27‐hydroxycholesterol [80]	20S‐hydroxycholesterol, 22R‐hydroxycholesterol, 24(S)‐hydroxycholesterol>25‐hydroxycholesterol, 27‐hydroxycholesterol [80]
Endogenous agonists	–	lanosterol (pEC_50_ 6) [114] – Mouse	–	–
Selective agonists	GW4064 (pEC_50_ 7.8) [95], obeticholic acid (pEC_50_ 7) [117], fexaramine (pEC_50_ 6.6) [36]	–	–	–
Selective antagonists	guggulsterone (pIC_50_ 5.7–6) [161]	–	–	–

### Comments

T0901317[123] and GW3965[28] are synthetic agonists acting at both LXRα and LXRβ with less than 10‐fold selectivity.

### Further Reading

A‐González N *et al*. (2011) Liver X receptors as regulators of macrophage inflammatory and metabolic pathways. *Biochim. Biophys. Acta* **1812**: 982‐94 [PMID:21193033]

Calkin AC *et al*. (2010) Liver x receptor signaling pathways and atherosclerosis. *Arterioscler. Thromb. Vasc. Biol.* **30**: 1513‐8 [PMID:20631351]

Chen WD *et al*. (2011) Nuclear bile acid receptor FXR in the hepatic regeneration. *Biochim. Biophys. Acta* **1812**: 888‐92 [PMID:21167938]

Claudel T *et al*. (2011) Role of nuclear receptors for bile acid metabolism, bile secretion, cholestasis, and gallstone disease. *Biochim. Biophys. Acta* **1812**: 867‐78 [PMID:21194565]

El‐Hajjaji FZ *et al*. (2011) Liver X receptors, lipids and their reproductive secrets in the male. *Biochim. Biophys. Acta* **1812**: 974‐81 [PMID:21334438]

Gadaleta RM *et al*. (2010) Bile acids and their nuclear receptor FXR: Relevance for hepatobiliary and gastrointestinal disease. *Biochim. Biophys. Acta* **1801**: 683‐92 [PMID:20399894]

Gardmo C *et al*. (2011) Proteomics for the discovery of nuclear bile acid receptor FXR targets. *Biochim. Biophys. Acta* **1812**: 836‐41 [PMID:21439373]

Kemper JK. (2011) Regulation of FXR transcriptional activity in health and disease: Emerging roles of FXR cofactors and post‐translational modifications. *Biochim. Biophys. Acta* **1812**: 842‐50 [PMID:21130162]

Matsubara T *et al*. (2013) FXR signaling in the enterohepatic system. *Mol. Cell. Endocrinol.* **368**: 17‐29 [PMID:22609541]

Moore DD *et al*. (2006) International Union of Pharmacology. LXII. The NR1H and NR1I receptors: constitutive androstane receptor, pregnene X receptor, farnesoid X receptor alpha, farnesoid X receptor beta, liver X receptor alpha, liver X receptor beta, and vitamin D receptor. *Pharmacol. Rev.* **58**: 742‐59 [PMID:17132852]

## 1I. Vitamin D receptor‐like receptors

### Overview

Vitamin D (VDR), Pregnane X (PXR) and Constitutive Androstane (CAR) receptors (**nomenclature as agreed by the** **NC‐IUPHAR** **Subcommittee on Nuclear Hormone Receptors**[**105**]) are members of the NR1I family of nuclear receptors, which form heterodimers with members of the retinoid X receptor family. PXR and CAR are activated by a range of exogenous compounds, with no established endogenous physiological agonists, although high concentrations of bile acids and bile pigments activate PXR and CAR [105].

**Table:** 

Nomenclature	Vitamin D receptor	Pregnane X receptor	Constitutive androstane receptor
Systematic nomenclature	NR1I1	NR1I2	NR1I3
HGNC, UniProt	VDR, P11473	NR1I2, O75469	NR1I3, Q14994
Endogenous agonists	1,25‐dihydroxyvitamin D3 (p*K*_d_ 8.9–9.2) [12, 39]	17β‐estradiol (Selective) [64]	–
Selective agonists	seocalcitol (p*K*_d_ 9.6) [29, 157], doxercalciferol	hyperforin (pEC_50_ 7.6) [106, 156], 5β‐pregnane‐3,20‐dione (pIC_50_ 6.4) [64], lovastatin (pEC_50_ 5.3–6) [82], rifampicin (pEC_50_ 5.5–6) [16, 82]	TCPOBOP (pEC_50_ 7.7) [149] – Mouse, CITCO (pEC_50_ 7.3) [92]
Selective antagonists	TEI‐9647 (pIC_50_ 8.2) [128] – Chicken, ZK159222 (pIC_50_ 7.5) [42, 60]	–	–
Comments	–	–	clotrimazole [107] and T0901317 [69] although acting at other sites, function as antagonists of the constitutive androstane receptor.

### Further Reading

Bikle DD. (2011) Vitamin D: an ancient hormone. *Exp. Dermatol.* **20**: 7‐13 [PMID:21197695]

Campbell FC *et al*. (2010) The yin and yang of vitamin D receptor (VDR) signaling in neoplastic progression: operational networks and tissue‐specific growth control. *Biochem. Pharmacol.* **79**: 1‐9 [PMID:19737544]

Chen Y *et al*. (2012) Nuclear receptors in the multidrug resistance through the regulation of drug‐metabolizing enzymes and drug transporters. *Biochem. Pharmacol.* **83**: 1112‐26 [PMID:22326308]

Cheng J *et al*. (2012) Pregnane X receptor as a target for treatment of inflammatory bowel disorders. *Trends Pharmacol. Sci.* **33**: 323‐30 [PMID:22609277]

Ihunnah CA *et al*. (2011) Nuclear receptor PXR, transcriptional circuits and metabolic relevance. *Biochim. Biophys. Acta* **1812**: 956‐63 [PMID:21295138]

Kachaylo EM *et al*. (2011) Constitutive androstane receptor (CAR) is a xenosensor and target for therapy. *Biochemistry Mosc.* **76**: 1087‐97 [PMID:22098234]

Moore DD *et al*. (2006) International Union of Pharmacology. LXII. The NR1H and NR1I receptors: constitutive androstane receptor, pregnene X receptor, farnesoid X receptor alpha, farnesoid X receptor beta, liver X receptor alpha, liver X receptor beta, and vitamin D receptor. *Pharmacol. Rev.* **58**: 742‐59 [PMID:17132852]

Plum LA *et al*. (2010) Vitamin D, disease and therapeutic opportunities. *Nat Rev Drug Discov* **9**: 941‐55 [PMID:21119732]

Staudinger JL *et al*. (2013) Nuclear‐receptor‐mediated regulation of drug‐ and bile‐acid‐transporter proteins in gut and liver. *Drug Metab. Rev.* **45**: 48‐59 [PMID:23330541]

## 2A. Hepatocyte nuclear factor‐4 receptors

### Overview

The nomenclature of hepatocyte nuclear factor‐4 receptors is agreed by the **NC‐IUPHAR** **Subcommittee on Nuclear Hormone Receptors** [**7**]. While linoleic acid has been identified as the endogenous ligand for HNF4α its function remains ambiguous [167]. HNF4γ has yet to be paired with an endogenous ligand.

**Table:** 

Nomenclature	Hepatocyte nuclear factor‐4‐α	Hepatocyte nuclear factor‐4‐γ
Systematic nomenclature	NR2A1	NR2A2
HGNC, UniProt	HNF4A, P41235	HNF4G, Q14541
Endogenous agonists	linoleic acid (Selective) [167]	–
Selective antagonists	BI6015 [72]	–
Comments	HNF4α has constitutive transactivation activity [167] and binds DNA as a homodimer [63].	–

### Further Reading

Benoit G *et al*. (2006) International Union of Pharmacology. LXVI. Orphan nuclear receptors. *Pharmacol. Rev.* **58**: 798‐836 [PMID:17132856]

Germain P *et al*. (2006) Overview of nomenclature of nuclear receptors. *Pharmacol. Rev.* **58**: 685‐704 [PMID:17132848]

Huang P *et al*. (2010) Structural overview of the nuclear receptor superfamily: insights into physiology and therapeutics. *Annu. Rev. Physiol.* **72**: 247‐72 [PMID:20148675]

Hwang‐Verslues WW *et al*. (2010) HNF4α–role in drug metabolism and potential drug target? *Curr Opin Pharmacol* **10**: 698‐705 [PMID:20833107]

Sladek FM. (2011) What are nuclear receptor ligands? *Mol. Cell. Endocrinol.* **334**: 3‐13 [PMID:20615454]

## 2B. Retinoid X receptors

### Overview

Retinoid X receptors (**nomenclature as agreed by the** **NC‐IUPHAR** **Subcommittee on Nuclear Hormone Receptors** [**45**]) are NR2B family members activated by alitretinoin and the RXR‐selective agonists bexarotene and LG100268, sometimes referred to as rexinoids. UVI3003[109] and HX 531 [37] have been described as a pan‐RXR antagonists. These receptors form RXR‐RAR heterodimers and RXR‐RXR homodimers [23, 97].

**Table:** 

Nomenclature	Retinoid X receptor‐α	Retinoid X receptor‐β	Retinoid X receptor‐γ
Systematic nomenclature	NR2B1	NR2B2	NR2B3
HGNC, UniProt	RXRA, P19793	RXRB, P28702	RXRG, P48443
(Sub)family‐selective agonists	bexarotene (pIC_50_ 7.4) [17, 22, 146]	bexarotene (pIC_50_ 7.7) [17, 22, 146]	bexarotene (pIC_50_ 7.5) [17, 22, 146]
Selective agonists	CD3254 (pIC_50_ 8.5) [48]	–	–

### Further Reading

Bour G *et al*. (2007) Protein kinases and the proteasome join in the combinatorial control of transcription by nuclear retinoic acid receptors. *Trends Cell Biol.* **17**: 302‐9 [PMID:17467991]

Duong V *et al*. (2011) The molecular physiology of nuclear retinoic acid receptors. From health to disease. *Biochim. Biophys. Acta* **1812**: 1023‐31 [PMID:20970498]

Germain P *et al*. (2006) International Union of Pharmacology. LXIII. Retinoid X receptors. *Pharmacol. Rev.* **58**: 760‐72 [PMID:17132853]

Lefebvre P *et al*. (2010) Retinoid X receptors: common heterodimerization partners with distinct functions. *Trends Endocrinol. Metab.* **21**: 676‐83 [PMID:20674387]

Maden M. (2007) Retinoic acid in the development, regeneration and maintenance of the nervous system. *Nat. Rev. Neurosci.* **8**: 755‐65 [PMID:17882253]

Mark M *et al*. (2006) Function of retinoid nuclear receptors: lessons from genetic and pharmacological dissections of the retinoic acid signaling pathway during mouse embryogenesis. *Annu. Rev. Pharmacol. Toxicol.* **46**: 451‐80 [PMID:16402912]

Pérez E *et al*. (2011) Modulation of RXR function through ligand design. *Biochim Biophys Acta* [PMID:21515403]

## 2C. Testicular receptors

### Overview

Testicular receptors (**nomenclature as agreed by the** **NC‐IUPHAR** **Subcommittee on Nuclear Hormone Receptors** [**7**]) have yet to be officially paired with an endogenous ligand, although testicular receptor 4 has been reported to respond to retinoids.

**Table:** 

Nomenclature	Testicular receptor 2	Testicular receptor 4
Systematic nomenclature	NR2C1	NR2C2
HGNC, UniProt	NR2C1, P13056	NR2C2, P49116
Endogenous agonists	–	retinol (Selective) [173], tretinoin (Selective) [173]
Comments	Forms a heterodimer with TR4; gene disruption appears without effect on testicular development or function [135].	Forms a heterodimer with TR2.

### Further Reading

Benoit G *et al*. (2006) International Union of Pharmacology. LXVI. Orphan nuclear receptors. *Pharmacol. Rev.* **58**: 798‐836 [PMID:17132856]

Duez H *et al*. (2009) Rev‐erb‐alpha: an integrator of circadian rhythms and metabolism. *J. Appl. Physiol.* **107**: 1972‐80 [PMID:19696364]

Germain P *et al*. (2006) Overview of nomenclature of nuclear receptors. *Pharmacol. Rev.* **58**: 685‐704 [PMID:17132848]

Huang P *et al*. (2010) Structural overview of the nuclear receptor superfamily: insights into physiology and therapeutics. *Annu. Rev. Physiol.* **72**: 247‐72 [PMID:20148675]

Phelan CA *et al*. (2010) Structure of Rev‐erbalpha bound to N‐CoR reveals a unique mechanism of nuclear receptor‐co‐repressor interaction. *Nat. Struct. Mol. Biol.* **17**: 808‐14 [PMID:20581824]

Schimmer BP *et al*. (2010) Minireview: steroidogenic factor 1: its roles in differentiation, development, and disease. *Mol. Endocrinol.* **24**: 1322‐37 [PMID:20203099]

Sladek FM. (2011) What are nuclear receptor ligands? *Mol. Cell. Endocrinol.* **334**: 3‐13 [PMID:20615454]

Yin L *et al*. (2010) Nuclear receptor Rev‐erbalpha: a heme receptor that coordinates circadian rhythm and metabolism. *Nucl Recept Signal* **8**: e001 [PMID:20414452]

Zhang Y *et al*. (2011) Role of nuclear receptor SHP in metabolism and cancer. *Biochim. Biophys. Acta* **1812**: 893‐908 [PMID:20970497]

Zhao Y *et al*. (2010) NR4A orphan nuclear receptors: transcriptional regulators of gene expression in metabolism and vascular biology. *Arterioscler. Thromb. Vasc. Biol.* **30**: 1535‐41 [PMID:20631354]

## 2E. Tailless‐like receptors

### Overview

Tailless‐like receptors (**nomenclature as agreed by the** **NC‐IUPHAR** **Subcommittee on Nuclear Hormone Receptors** [**7**]) have yet to be officially paired with an endogenous ligand.

**Table:** 

Nomenclature	TLX	PNR
Systematic nomenclature	NR2E1	NR2E3
HGNC, UniProt	NR2E1, Q9Y466	NR2E3, Q9Y5X4
Comments	Gene disruption is associated with abnormal brain development [76, 104].	–

### Further Reading

Benoit G *et al*. (2006) International Union of Pharmacology. LXVI. Orphan nuclear receptors. *Pharmacol. Rev.* **58**: 798‐836 [PMID:17132856]

Germain P *et al*. (2006) Overview of nomenclature of nuclear receptors. *Pharmacol. Rev.* **58**: 685‐704 [PMID:17132848]

Gui H *et al*. (2011) A tale of tailless. *Dev. Neurosci.* **33**: 1‐13 [PMID:21124006]

Huang P *et al*. (2010) Structural overview of the nuclear receptor superfamily: insights into physiology and therapeutics. *Annu. Rev. Physiol.* **72**: 247‐72 [PMID:20148675]

Sladek FM. (2011) What are nuclear receptor ligands? *Mol. Cell. Endocrinol.* **334**: 3‐13 [PMID:20615454]

## 2F. COUP‐TF‐like receptors

### Overview

COUP‐TF‐like receptors (**nomenclature as agreed by the** **NC‐IUPHAR** **Subcommittee on Nuclear Hormone Receptors** [**7**]) have yet to be officially paired with an endogenous ligand.

**Table:** 

Nomenclature	COUP‐TF1	COUP‐TF2	V‐erbA‐related gene
Systematic nomenclature	NR2F1	NR2F2	NR2F6
HGNC, UniProt	NR2F1, P10589	NR2F2, P24468	NR2F6, P10588
Comments	Gene disruption is perinatally lethal [121].	Gene disruption is embryonically lethal [118].	Gene disruption impairs CNS development [155].

### Further Reading

Benoit G *et al*. (2006) International Union of Pharmacology. LXVI. Orphan nuclear receptors. *Pharmacol. Rev.* **58**: 798‐836 [PMID:17132856]

Germain P *et al*. (2006) Overview of nomenclature of nuclear receptors. *Pharmacol. Rev.* **58**: 685‐704 [PMID:17132848]

Huang P *et al*. (2010) Structural overview of the nuclear receptor superfamily: insights into physiology and therapeutics. *Annu. Rev. Physiol.* **72**: 247‐72 [PMID:20148675]

Lin FJ *et al*. (2011) Coup d'Etat: an orphan takes control. *Endocr. Rev.* **32**: 404‐21 [PMID:21257780]

Sladek FM. (2011) What are nuclear receptor ligands? *Mol. Cell. Endocrinol.* **334**: 3‐13 [PMID:20615454]

## 3B. Estrogen‐related receptors

### Overview

Estrogen‐related receptors (**nomenclature as agreed by the** **NC‐IUPHAR** **Subcommittee on Nuclear Hormone Receptors** [**7**]) have yet to be officially paired with an endogenous ligand.

**Table:** 

Nomenclature	Estrogen‐related receptor‐α	Estrogen‐related receptor‐β	Estrogen‐related receptor‐γ
Systematic nomenclature	NR3B1	NR3B2	NR3B3
HGNC, UniProt	ESRRA, P11474	ESRRB, O95718	ESRRG, P62508
Comments	Activated by some dietary flavonoids [141]; activated by the syntheticagonist GSK4716 [176] and blocked by XCT790 [160].	May be activated by DY131 [166].	May be activated by DY131 [166].

### Further Reading

Benoit G *et al*. (2006) International Union of Pharmacology. LXVI. Orphan nuclear receptors. *Pharmacol. Rev.* **58**: 798‐836 [PMID:17132856]

Deblois G *et al*. (2011) Functional and physiological genomics of estrogen‐related receptors (ERRs) in health and disease. *Biochim. Biophys. Acta* **1812**: 1032‐40 [PMID:21172432]

Deblois G *et al*. (2013) Oestrogen‐related receptors in breast cancer: control of cellular metabolism and beyond. *Nat. Rev. Cancer* **13**: 27‐36 [PMID:23192231]

Germain P *et al*. (2006) Overview of nomenclature of nuclear receptors. *Pharmacol. Rev.* **58**: 685‐704 [PMID:17132848]

Huang P *et al*. (2010) Structural overview of the nuclear receptor superfamily: insights into physiology and therapeutics. *Annu. Rev. Physiol.* **72**: 247‐72 [PMID:20148675]

Hwang‐Verslues WW *et al*. (2010) HNF4α–role in drug metabolism and potential drug target? *Curr Opin Pharmacol* **10**: 698‐705 [PMID:20833107]

Sladek FM. (2011) What are nuclear receptor ligands? *Mol. Cell. Endocrinol.* **334**: 3‐13 [PMID:20615454]

## 4A. Nerve growth factor IB‐like receptors

### Overview

Nerve growth factor IB‐like receptors (**nomenclature as agreed by the** **NC‐IUPHAR** **Subcommittee on Nuclear Hormone Receptors** [**7**]) have yet to be officially paired with an endogenous ligand.

**Table:** 

Nomenclature	Nerve Growth factor IB	Nuclear receptor related 1	Neuron‐derived orphan receptor 1
Systematic nomenclature	NR4A1	NR4A2	NR4A3
HGNC, UniProt	NR4A1, P22736	NR4A2, P43354	NR4A3, Q92570
Comments	An endogenous agonist, cytosporone B, has been described [168], although structural analysis and molecular modelling has not identified a ligand binding site [4, 40, 154].	–	–

### Further Reading

Benoit G *et al*. (2006) International Union of Pharmacology. LXVI. Orphan nuclear receptors. *Pharmacol. Rev.* **58**: 798‐836 [PMID:17132856]

Germain P *et al*. (2006) Overview of nomenclature of nuclear receptors. *Pharmacol. Rev.* **58**: 685‐704 [PMID:17132848]

Huang P *et al*. (2010) Structural overview of the nuclear receptor superfamily: insights into physiology and therapeutics. *Annu. Rev. Physiol.* **72**: 247‐72 [PMID:20148675]

McMorrow JP *et al*. (2011) Inflammation: a role for NR4A orphan nuclear receptors? *Biochem. Soc. Trans.* **39**: 688‐93 [PMID:21428963]

Mohan HM *et al*. (2012) Molecular pathways: the role of NR4A orphan nuclear receptors in cancer. *Clin. Cancer Res.* **18**: 3223‐8 [PMID:22566377]

Pearen MA *et al*. (2010) Minireview: Nuclear hormone receptor 4A signaling: implications for metabolic disease. *Mol. Endocrinol.* **24**: 1891‐903 [PMID:20392876]

Sladek FM. (2011) What are nuclear receptor ligands? *Mol. Cell. Endocrinol.* **334**: 3‐13 [PMID:20615454]

Zhao Y *et al*. (2010) NR4A orphan nuclear receptors: transcriptional regulators of gene expression in metabolism and vascular biology. *Arterioscler. Thromb. Vasc. Biol.* **30**: 1535‐41 [PMID:20631354]

van Tiel CM *et al*. (2012) NR4All in the vessel wall. *J. Steroid Biochem. Mol. Biol.* **130**: 186‐93 [PMID:21277978]

## 5A. Fushi tarazu F1‐like receptors

### Overview

Fushi tarazu F1‐like receptors (**nomenclature as agreed by the** **NC‐IUPHAR** **Subcommittee on Nuclear Hormone Receptors** [**7**]) have yet to be officially paired with an endogenous ligand.

**Table:** 

Nomenclature	Steroidogenic factor 1	Liver receptor homolog‐1
Systematic nomenclature	NR5A1	NR5A2
HGNC, UniProt	NR5A1, Q13285	NR5A2, O00482
Comments	Reported to be inhibited by AC45594 [32] and SID7969543 [91].	–

### Further Reading

Benoit G *et al*. (2006) International Union of Pharmacology. LXVI. Orphan nuclear receptors. *Pharmacol. Rev.* **58**: 798‐836 [PMID:17132856]

Büdefeld T *et al*. (2012) Steroidogenic factor 1 and the central nervous system. *J. Neuroendocrinol.* **24**: 225‐35 [PMID:21668533]

El‐Khairi R *et al*. (2011) Role of DAX‐1 (NR0B1) and steroidogenic factor‐1 (NR5A1) in human adrenal function. *Endocr Dev* **20**: 38‐46 [PMID:21164257]

Fernandez‐Marcos PJ *et al*. (2011) Emerging actions of the nuclear receptor LRH‐1 in the gut. *Biochim. Biophys. Acta* **1812**: 947‐55 [PMID:21194563]

Ferraz‐de‐Souza B *et al*. (2011) Steroidogenic factor‐1 (SF‐1, NR5A1) and human disease. *Mol. Cell. Endocrinol.* **336**: 198‐205 [PMID:21078366]

Germain P *et al*. (2006) Overview of nomenclature of nuclear receptors. *Pharmacol. Rev.* **58**: 685‐704 [PMID:17132848]

Huang P *et al*. (2010) Structural overview of the nuclear receptor superfamily: insights into physiology and therapeutics. *Annu. Rev. Physiol.* **72**: 247‐72 [PMID:20148675]

Lazarus KA *et al*. (2012) Therapeutic potential of Liver Receptor Homolog‐1 modulators. *J. Steroid Biochem. Mol. Biol.* **130**: 138‐46 [PMID:22266285]

Mlnynarczuk J *et al*. (2010) The role of the orphan receptor SF‐1 in the development and function of the ovary. *Reprod Biol* **10**: 177‐93 [PMID:21113200]

Schimmer BP *et al*. (2010) Minireview: steroidogenic factor 1: its roles in differentiation, development, and disease. *Mol. Endocrinol.* **24**: 1322‐37 [PMID:20203099]

Sladek FM. (2011) What are nuclear receptor ligands? *Mol. Cell. Endocrinol.* **334**: 3‐13 [PMID:20615454]

## 6A. Germ cell nuclear factor receptors

### Overview

Germ cell nuclear factor receptors (**nomenclature as agreed by the** **NC‐IUPHAR** **Subcommittee on Nuclear Hormone Receptors** [**7**]) have yet to be officially paired with an endogenous ligand.

**Table:** 

Nomenclature	Germ cell nuclear factor
Systematic nomenclature	NR6A1
HGNC, UniProt	NR6A1, Q15406

### Further Reading

Benoit G *et al*. (2006) International Union of Pharmacology. LXVI. Orphan nuclear receptors. *Pharmacol. Rev.* **58**: 798‐836 [PMID:17132856]

Germain P *et al*. (2006) Overview of nomenclature of nuclear receptors. *Pharmacol. Rev.* **58**: 685‐704 [PMID:17132848]

Huang P *et al*. (2010) Structural overview of the nuclear receptor superfamily: insights into physiology and therapeutics. *Annu. Rev. Physiol.* **72**: 247‐72 [PMID:20148675]

Sladek FM. (2011) What are nuclear receptor ligands? *Mol. Cell. Endocrinol.* **334**: 3‐13 [PMID:20615454]

## 0B. DAX‐like receptors

### Overview

Dax‐like receptors (**nomenclature as agreed by the** **NC‐IUPHAR** **Subcommittee on Nuclear Hormone Receptors** [**7**]) have yet to be officially paired with an endogenous ligand.

**Table:** 

Nomenclature	DAX1	SHP
Systematic nomenclature	NR0B1	NR0B2
HGNC, UniProt	NR0B1, P51843	NR0B2, Q15466

### Further Reading

Benoit G *et al*. (2006) International Union of Pharmacology. LXVI. Orphan nuclear receptors. *Pharmacol. Rev.* **58**: 798‐836 [PMID:17132856]

Chanda D *et al*. (2008) Molecular basis of endocrine regulation by orphan nuclear receptor Small Heterodimer Partner. *Endocr. J.* **55**: 253‐68 [PMID:17984569]

Ehrlund A *et al*. (2012) Ligand‐independent actions of the orphan receptors/corepressors DAX‐1 and SHP in metabolism, reproduction and disease. *J. Steroid Biochem. Mol. Biol.* **130**: 169‐79 [PMID:21550402]

El‐Khairi R *et al*. (2011) Role of DAX‐1 (NR0B1) and steroidogenic factor‐1 (NR5A1) in human adrenal function. *Endocr Dev* **20**: 38‐46 [PMID:21164257]

Germain P *et al*. (2006) Overview of nomenclature of nuclear receptors. *Pharmacol. Rev.* **58**: 685‐704 [PMID:17132848]

Huang P *et al*. (2010) Structural overview of the nuclear receptor superfamily: insights into physiology and therapeutics. *Annu. Rev. Physiol.* **72**: 247‐72 [PMID:20148675]

Mlnynarczuk J *et al*. (2010) The role of the orphan receptor SF‐1 in the development and function of the ovary. *Reprod Biol* **10**: 177‐93 [PMID:21113200]

Sladek FM. (2011) What are nuclear receptor ligands? *Mol. Cell. Endocrinol.* **334**: 3‐13 [PMID:20615454]

Zhang Y *et al*. (2011) Role of nuclear receptor SHP in metabolism and cancer. *Biochim. Biophys. Acta* **1812**: 893‐908 [PMID:20970497]

## Steroid hormone receptors

### Overview

Steroid hormone receptors (**nomenclature as agreed by the** **NC‐IUPHAR** **Subcommittee on Nuclear Hormone Receptors** [**30**, **89**]) are nuclear hormone receptors of the NR3 class, with endogenous agonists that may be divided into 3‐hydroxysteroids (estrone and 17β‐estradiol) and 3‐ketosteroids (dihydrotestosterone[DHT], aldosterone, cortisol, corticosterone, progesterone and testosterone). These receptors exist as dimers coupled with chaperone molecules (such as hsp90β(*HSP90AB1*, P08238) and immunophilin FKBP52:*FKBP4*, Q02790), which are shed on binding the steroid hormone. Although rapid signalling phenomena are observed [84, 120], the principal signalling cascade appears to involve binding of the activated receptors to nuclear hormone response elements of the genome, with a 15‐nucleotide consensus sequence AGAACAnnnTGTTCT (*i.e.* an inverted palindrome) as homo‐ or heterodimers. They also affect transcription by protein‐protein interactions with other transcription factors, such as activator protein 1 (AP‐1) and nuclear factor *κ*B (NF‐*κ*B). Splice variants of each of these receptors can form functional or non‐functional monomers that can dimerize to form functional or non‐functional receptors. For example, alternative splicing of PR mRNA produces A and B monomers that combine to produce functional AA, AB and BB receptors with distinct characteristics [150].

A 7TM receptor responsive to estrogen (*GPER1*, Q99527, also known as GPR30, see [119]) has been described. Human orthologues of 7TM 'membrane progestin receptors' (*PAQR7*, *PAQR8* and *PAQR5*), initially discovered in fish [174, 175], appear to localize to intracellular membranes and respond to 'non‐genomic' progesterone analogues independently of G proteins [137].

## 3A. Estrogen receptors

### Overview

Estrogen receptor (ER) activity regulates diverse physiological processes *via* transcriptional modulation of target genes. The selection of target genes and the magnitude of the response, be it induction or repression, are determined by many factors, including the effect of the hormone ligand and DNA binding on ER structural conformation, and the local cellular regulatory environment. The cellular environment defines the specific complement of DNA enhancer and promoter elements present and the availability of coregulators to form functional transcription complexes. Together, these determinants control the resulting biological response.

**Table:** 

Nomenclature	Estrogen receptor‐α	Estrogen receptor‐β
Systematic nomenclature	NR3A1	NR3A2
HGNC, UniProt	ESR1, P03372	ESR2, Q92731
Endogenous agonists	estriol (p*K*_i_ 8.7) [75], estrone (p*K*_i_ 8.5) [75]	–
Selective agonists	propylpyrazoletriol (p*K*_i_ 9.6) [74, 138], ethinyl estradiol (pIC_50_ 8.7) [62]	WAY200070 (pIC_50_ 8.5–9) [94], diarylpropionitril (p*K*_i_ 8.6) [100, 138], prinaberel (pIC_50_ 8.3) [94]
(Sub)family‐selective antagonists	bazedoxifene (pIC_50_ 7.6) [103]	bazedoxifene (pIC_50_ 7.1) [103]
Selective antagonists	clomiphene (p*K*_i_ 8.9) [[3]], methyl‐piperidino‐pyrazole (p*K*_i_ 8.6) [142]	R,R‐THC (p*K*_i_ 8.4) [99, 143], PHTPP (p*K*_i_ 6.9) [172]

### Comments

R,R‐THC exhibits partial agonist activity at ERα[99, 143]. Estrogen receptors may be blocked non‐selectively by tamoxifen and raloxifene and labelled by [^3^H]17β‐estradiol and [^3^H]tamoxifen. Many agents thought initially to be antagonists at estrogen receptors appear to have tissue‐specific efficacy (*e.g*. Tamoxifen is an antagonist at estrogen receptors in the breast, but is an agonist at estrogen receptors in the uterus), hence the descriptor SERM (selective estrogen receptor modulator) or SnuRM (selective nuclear receptor modulator). Y134 has been suggested to be an ERα‐selective estrogen receptor modulator [112].

## 3C. 3‐Ketosteroid receptors

**Table:** 

Nomenclature	Androgen receptor	Glucocorticoid receptor	Mineralocorticoid receptor	Progesterone receptor
Systematic nomenclature	NR3C4	NR3C1	NR3C2	NR3C3
HGNC, UniProt	AR, P10275	NR3C1, P04150	NR3C2, P08235	PGR, P06401
Rank order of potency	dihydrotestosterone>testosterone	cortisol, corticosterone≫aldosterone, deoxycortisone [127]	corticosterone, cortisol, aldosterone, progesterone [127]	progesterone
Endogenous agonists	dihydrotestosterone (p*K*_d_ 9.3) [147]	–	aldosterone (Selective) (pIC_50_ 9.8–10) [58, 127]	progesterone (Selective) (pEC_50_ 8.8) [38]
Agonists	methyltestosterone (Androgen receptor promoter activity in luciferase reporter assay) (pEC_50_ 9.7) [1], danazol (p*K*_i_ 8) [24] – Rat, ethylestrenol, nandrolone	–	–	–
Selective agonists	testosterone propionate (p*K*_i_ 9.6) [96], mibolerone (pIC_50_ 9) [49], fluoxymesterone (p*K*_i_ 8.2) [61], methyltrienolone (pEC_50_<5) [152], dromostanolone propionate	fluticasone propionate (pIC_50_ 9.3) [11], clobetasol propionate (p*K*_i_ 9.2) [[3]], desoximetasone (p*K*_i_ 8.9) [[3]], fluorometholone (p*K*_i_ 8.8) [[3]], flunisolide (p*K*_i_ 8.6) [[3]], diflorasone diacetate (p*K*_i_ 8.5) [[3]], fluocinolone acetonide (pIC_50_ 8.5) [[3]], beclometasone (p*K*_i_ 8.4) [[3]], methylprednisolone (p*K*_i_ 8.3) [[3]], fluocinonide (pIC_50_ 8.3) [[3]], betamethasone (pIC_50_ 8.1) [[3]], budesonide (pEC_50_ 7.9) [102], triamcinolone (pIC_50_ 7.7) [87], ZK216348 (pIC_50_ 7.7) [132], ciclesonide (p*K*_i_ 7.4) [6], prednisone (p*K*_i_ 6.3) [[3]], RU26988 – Unknown, RU28362, difluprednate [145], fluticasone	–	medroxyprogesterone (Affinity at human PR‐A) (p*K*_i_ 9.5) [170], ORG2058, levonorgestrel [10, 130]
Antagonists	cyproterone acetate (p*K*_i_ 7.8) [55]	mifepristone (p*K*_d_ 9.4) [57, 127]	nimodipine (inhibition of aldosterone‐induced luciferase activity in a reporter system driven by the mineralocorticoid receptor ligand binding domain) (pIC_50_ 6.8) [34]	–
Selective antagonists	bicalutamide (p*K*_i_ 7.7) [71], PF0998425 (pIC_50_ 7.1–7.5) [86], enzalutamide (pIC_50_ 7.4) [148], nilutamide (pIC_50_ 7.1–7.1) [136], hydroxyflutamide (pEC_50_ 6.6) [152], galeterone (pIC_50_ 6.4) [56], flutamide (Displacement of ^3^[H] testosterone from wild‐type androgen receptors) (p*K*_i_ 5.4) [151]	onapristone (pIC_50_ 7.6) [169], ZK112993	finerenone (pIC_50_ 7.7) [21], eplerenone (p*K*_i_ 6.9) [5], onapristone (pIC_50_ 6.3) [169], RU28318, ZK112993	ulipristal acetate (pIC_50_ 9.7) [124], mifepristone (Mixed) (p*K*_i_ 9) [171], onapristone (p*K*_i_ 7.7) [54], ZK112993
Labelled ligands	[^3^H]dihydrotestosterone (Selective Agonist), [^3^H]methyltrienolone (Selective Agonist), [^3^H]mibolerone (Agonist)	[^3^H]dexamethasone (Agonist)	[^3^H]aldosterone (Selective Agonist) (p*K*_d_ 9.5–9.4) [44, 140] – Rat	[^3^H]ORG2058 (Selective Agonist)

### Comments

[^3^H]dexamethasone also binds to MR*in vitro*. PR antagonists have been suggested to subdivide into Type I (*e.g.* onapristone) and Type II (*e.g*. ZK112993) groups. These groups appear to promote binding of PR to DNA with different efficacies and evoke distinct conformational changes in the receptor, leading to a transcription‐neutral complex [43, 83]. Mutations in AR underlie testicular feminization and androgen insensibility syndromes, spinal and bulbar muscular atrophy (Kennedy's disease).
